# Trial to Encourage Adoption and Maintenance of a Mediterranean Diet (TEAM-MED): Protocol for a Randomised Feasibility Trial of a Peer Support Intervention for Dietary Behaviour Change in Adults at High Cardiovascular Disease Risk

**DOI:** 10.3390/ijerph15061130

**Published:** 2018-05-31

**Authors:** Claire T. McEvoy, Sarah E. Moore, Katherine M. Appleton, Margaret E. Cupples, Christina M. Erwin, Steven J. Hunter, Frank Kee, David McCance, Christopher C. Patterson, Ian S. Young, Michelle C. McKinley, Jayne V. Woodside

**Affiliations:** 1 Centre for Public Health, Queen’s University Belfast, Grosvenor Road, Belfast BT12 6BJ, UK; sarah.moore@qub.ac.uk (S.E.M.); M.Cupples@qub.ac.uk (M.E.C.); Cerwin01@qub.ac.uk (C.M.E.); F.Kee@qub.ac.uk (F.K.); C.Patterson@qub.ac.uk (C.C.P.); I.Young@qub.ac.uk (I.S.Y.); m.mckinley@qub.ac.uk (M.C.M.); j.woodside@qub.ac.uk (J.V.W.); 2 Department of Psychology, Faculty of Science and Technology, Bournemouth University, Fern Barrow, Talbot Campus, Bournemouth BH12 5BB, UK; k.appleton@bournemouth.ac.uk; 3 UK Clinical Research Collaboration, Centre of Excellence for Public Health, Queen’s University Belfast, Grosvenor Road, Belfast BT12 6BJ, UK; 4 Belfast Health and Social Care Trust, Diabetes and Endocrinology, Grosvenor Road, Belfast BT12 6BA, UK; Steven.hunter@belfasttrust.hscni.net (S.J.H.); David.mccance@belfasttrust.hscni.net (D.M.)

**Keywords:** Mediterranean diet, behaviour change, peer support, cardiovascular disease, public health

## Abstract

Adoption of a Mediterranean diet (MD) reduces cardiovascular disease (CVD) risk. However, interventions to achieve dietary behaviour change are typically resource intensive. Peer support offers a potentially low-cost approach to encourage dietary change. The primary objective of this randomised controlled trial is to explore the feasibility of peer support versus a previously tested dietetic-led intervention to encourage MD behaviour change, and to test recruitment strategies, retention and attrition in order to inform the design of a definitive trial. A total of 75 overweight adults at high CVD risk who do not follow a MD (Mediterranean Diet Score (MDS ≤ 3)) will be randomly assigned to either: a minimal intervention (written materials), a proven intervention (dietetic support, written materials and key MD foods), or a peer support intervention (group-based community programme delivered by lay peers) for 12 months. The primary end-point is change in MDS from baseline to 6 months (adoption of MD). Secondary end-points include: change in MDS from 6 to 12 months (maintenance of MD), effects on nutritional biomarkers and CVD risk factors, fidelity of implementation, acceptability and feasibility of the peer support intervention. This study will generate important data regarding the feasibility of peer support for ease of adoption of MD in an ‘at risk’ Northern European population. Data will be used to direct a larger scale trial, where the clinical efficacy and cost-effectiveness of peer support will be tested.

## 1. Introduction

### 1.1. The Cardioprotective Benefits of a Mediterranean Diet

The traditional Mediterranean diet (MD), rich in fruit, vegetables, wholegrains, nuts, fish and olive oil, and low in red meat and processed foods [1], is rated as the most likely dietary pattern to offer protection against cardiovascular disease (CVD) [2]. This is supported by robust evidence from epidemiologic studies [3,4,5], which have been subjected to meta-analysis [6]. Furthermore, the evidence base is strengthened by the addition of randomised controlled trials (RCT) demonstrating a clear benefit of increased MD adherence for primary and secondary CVD prevention. The Lyon Diet Heart Study in patients with myocardial infarction, showed a significant 50–70% reduction in CVD events in the MD intervention group compared with standard care [7]. The PREDIMED (Prevención con Dieta Mediterránea) primary prevention trial, involving 7447 adults at high CVD risk, reported a significant 30% reduction in CVD events in those consuming a MD supplemented with either olive oil or nuts, compared to those consuming a low-fat control diet [8]. The cardio-protective mechanisms of the diet are not fully understood; however, increased adherence appears to modify established vascular risk factors including blood lipids, blood pressure, insulin resistance and inflammation [9,10]. Emerging evidence also suggests that adopting a MD can have additional benefits for overall longevity and chronic conditions, such as diabetes, cancer and Alzheimer’s disease [6]. The health benefits offered by a MD appear to be attributable to biological interactions between different food components rather than the effects of single nutrients [11]. Hence, this dietary pattern has been proposed as a palatable and beneficial lifestyle change [12] and has been recommended as a healthy eating pattern in the 2015–2020 dietary guidelines for Americans [13]. However, accessibility to foods and sociocultural influences on food choice differ between countries and populations [14] and it is not yet clear whether non-Mediterranean countries can adopt and maintain dietary behaviours consistent with a traditional MD. 

### 1.2. Peer Support as a Potential Strategy to Encourage Dietary Behaviour Change

Successful dietary change toward a MD is shown to be possible with resource intensive interventions, usually delivered by health professionals [8,12,15,16]. Interventions of this nature may be challenging to scale-up for wider public health benefit. One solution is to involve lay peers in the delivery of dietary behaviour change programmes. This type of organised peer support offers an alternative and potentially low-cost way of promoting dietary change [17] and capitalises on social support networks between individuals to strengthen factors associated with successful behaviour change, such as knowledge, self-efficacy and resilience [18,19]. 

There is a large body of literature on peer support for self-management of health behaviours and disease conditions. However, the effectiveness of peer support as a sole method for changing dietary behaviour is not entirely consistent. Some peer support interventions have demonstrated significant improvements in diet quality [20,21,22,23,24] while other interventions show no effect [25,26]. Similarly, systematic reviews of the effectiveness of peer support to improve vascular and diabetes outcomes have been unable to draw firm conclusions [27,28]. The effectiveness of peer support interventions is difficult to determine owing to substantial between study heterogeneity in study design, study population, sample size and outcome measures. In addition, there is considerable variation in the format, content and intensity of peer support provided, with few studies reporting behavioural strategies used to encourage behaviour change or the fidelity of intervention delivery. Overall, peer support could be a promising strategy for promoting MD behaviour change [16] but further well-designed trials are required to determine effectiveness. 

### 1.3. Development of a Tailored Peer Support Intervention

Evidence suggests that lifestyle interventions informed by theoretically-driven behaviour change models are more successful and lead to stronger and more sustained changes [29]. Therefore, in accordance with the Medical Research Council framework for developing and evaluating complex interventions to improve health [30] and the Behaviour Change Wheel [31], we developed a theory-based, tailored peer support intervention delivered in a community setting to encourage dietary behaviour change in a Northern European population at high CVD risk. The process used to develop the intervention involved both a literature review [32,33] and qualitative research with our target high CVD risk population [34,35]. The intervention is underpinned by the social support theoretical model, defined as ‘the process through which social relationships might promote health and well-being’ [19]. Within this model, we postulated that the peer support intervention would provide informational, emotional and appraisal support to encourage dietary change towards a MD. We also incorporated psychological theories of behaviour change at the interpersonal e.g., Social Cognitive Theory, and intrapersonal level e.g., Health Beliefs Model. It is unclear how peer support can change dietary behaviour towards a MD, however potential mechanisms are likely to be via strengthening of variables known to support behaviour change [18], for example, social support, self-efficacy, self-regulation, problem-solving, knowledge, attitudes and skill acquisition. We incorporated strategies in the intervention to enhance these variables based on the Capability, Opportunity, Motivation, Behaviour (COM-B) model to specifically address identified barriers to adopting a MD in our target population [31,34].

### 1.4. Aim

This paper describes the protocol for the TEAM-MED study (Trial to Encourage Adoption and Maintenance of a MEiterranean Diet) that aims to explore the feasibility of peer support versus a previously tested dietitian-led intervention to encourage MD behaviour change [8], and to test recruitment strategies, retention and attrition, to inform the design of a definitive trial where the clinical and cost-effectiveness of the peer support intervention will be tested.

## 2. Materials and Methods

### 2.1. TEAM-MED Objectives

The objectives to meet the study aim are to:

- Estimate and compare the variability of Mediterranean Diet Score (MDS) from baseline to 6 months and from 6 months to 12 months between the peer support intervention and other intervention groups
- Estimate and compare the variability of biochemical markers of nutritional status and health markers over the course of the intervention, as for MDS, between the peer support intervention and other intervention groups
- Test recruitment strategies, retention, attrition rates
- Estimate the sample size for a large-scale trial

Methods to achieve these objectives are described in detail below. Further study objectives related to the planned process evaluation (to be published separately) are to:

- Test the validity of the theoretical model underpinning the peer support intervention
- Evaluate the training and support provided to peer supporters to deliver the intervention
- Determine fidelity of implementation and acceptability of the intervention
- Assess outcome data collection processes within the pilot trial, including those to explore mediators and moderators of MD adherence and cost-effectiveness
- Explore reasons for withdrawal for study participants and peer supporters recruited to deliver the peer support intervention

### 2.2. Design

TEAM-MED is a 12 month pilot parallel group RCT designed to evaluate the feasibility of a community-based peer support intervention to encourage adoption and maintenance of a MD, in comparison to a proven dietetic-led intervention [8] and a minimal intervention (control group), in adults at high CVD risk in Northern Ireland. Ethical approval has been received from the Office for Research Ethics Committees Northern Ireland (HSC RECA; ref 13/NI/0152) who will also review any changes made to the protocol. The study protocol is registered on ControlledTrials.com (ID no. ISRCTN68779848). Informed written consent for study procedures and collection, handling and storage of biological samples will be obtained from all study participants by the study researcher. 

**Figure 1:**
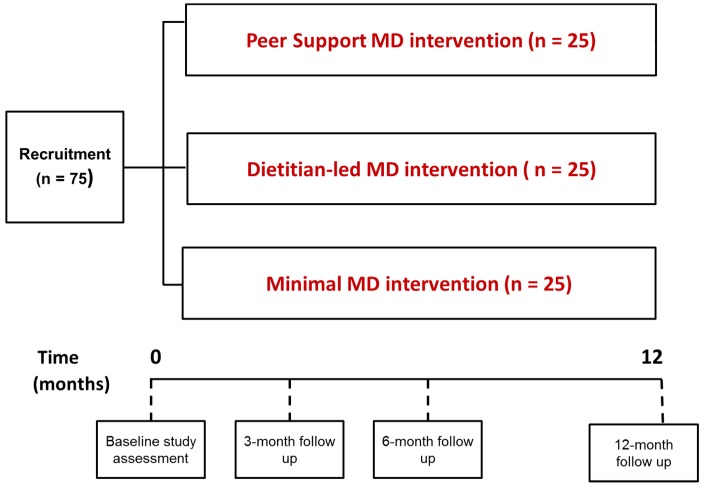
Overview of Trial to Encourage Adoption and Maintenance of a MEditerranean Diet (TEAM-MED) study design. MD: Mediterranean diet.

An overview of the study design is shown in Figure 1 and a summary is reported using the SPIRIT checklist (Table S1). Following a screening appointment, eligible participants will be block randomized (using a computer-generated random-number sequence) to either: (i) a peer support intervention; (ii) a dietetic-led intervention; or, (iii) a minimal intervention. Participants will be informed of their allocated group by the researcher after baseline data is collected. It will not be possible to blind study researchers or participants to the allocated intervention group, however, laboratory and data analysis and assessment of primary outcome will be carried out by an investigator blinded to treatment allocation. 

The three TEAM-MED intervention groups (peer support, dietitian-led support and minimal support) as discussed in Section 2.4, vary in the intensity and nature of support provided to participants to encourage adoption of dietary behaviours consistent with a MD. The target MD behaviours for this study are based on the PREDIMED study [8] adapted for the current population [34] and include: preferential consumption of wholegrain cereal foods, fruits ≥ 2 portions (160 g)/day, vegetables ≥ 3 portions (240 g)/day, olive/rapeseed oils ≥ 4 tbsp. (60 mL)/day and/or Monounsaturated Fatty Acid (MUFA)-rich spreads ≥ 3 tsp. (15 g)/day, nuts ≥ 3 handfuls (90 g)/week, legumes ≥ 3 portions (240 g)/week, fish ≥ 3 servings (420 g)/week, red meat ≤ 2 servings (300 g)/week, processed meat ≤ 1 serving (150 g)/week, confectionary ≤ 3 servings/week, alcohol (if consumed) 125–375 mL ≥ 3 days/week.

The inclusion of rapeseed oil as an alternative to olive oil was a pragmatic decision taken to address specific barriers to olive oil intake e.g., cost, taste and cultural differences in food choice that were identified in our target population [32]. Rapeseed oil is a more familiar culinary oil with a similar fatty acid profile albeit with lower polyphenol and bioactive content compared to olive oil. 

Specific behaviour change techniques derived from published taxonomies [36,37] are incorporated in each TEAM-MED intervention to promote MD behaviour change, as shown in Table 1. The duration of the intervention is one year and study assessments will be conducted at baseline and at 3-, 6- and 12-month follow up. 

### 2.3. Study Participants

#### 2.3.1. Participant Recruitment

We aim to recruit 75 male/female participants aged 40 years or more from hospital outpatient clinics, GP surgeries and the general population. Potential participants will attend a dedicated research facility at Queen’s University Belfast for a 45-min screening appointment conducted by a study researcher. A questionnaire to record demographics, health and medical history (including medications and supplement usage) and stage of dietary change towards a MD [38], will be used together with CVD risk prediction charts [39] and an assessment of participant’s baseline MD adherence using an existing 14-item questionnaire from the PREDIMED study [8] and modified to reflect the Northern European diet to assess study eligibility. 

#### 2.3.2. Inclusion Criteria

Participants will be considered eligible to enrol in the study if they are overweight (BMI > 27 and ≤ 45 kg/m^2^), aged 40 years or more, have low adherence to a MD (MDS ≤ 3) and a combination of risk factors which places them at high total risk (estimated multifactorial CVD risk ≥ 20% over ten years) of developing atherosclerotic CVD for the first time. 

#### 2.3.3. Exclusion Criteria

Participants will not be eligible to enrol in the study if they have established diabetes mellitus, CVD or a medical condition or dietary restriction(s) that would substantially limit their ability to complete the study requirements, have an excessive alcohol consumption (>28 Units/week men or >21 Units/week women), have a low predicted likelihood to change dietary habits or are unable to provide informed consent. 

#### 2.3.4. Peer Supporters

Peer supporters (lay participants and/or community health workers) will be recruited from the general public and from local community networks, volunteer websites and health centres. Two peer supporters will be allocated to each group formed within the peer support intervention. It is envisaged that one peer supporter will have been successful in making positive changes to their diet and the other will be familiar with community networks and group facilitation. Potential peer supporters will undergo study eligibility screening similar to that described for study participants above, and an additional screening interview, conducted by two members of the study team, to determine their suitability and commitment to deliver the peer support intervention. In total, 12 peer supporters will be recruited and trained, which is more than required to successfully deliver the proposed intervention. However, access to a pool of trained peer supporters will allow any setbacks in study implementation to be quickly addressed, for example, withdrawal of peer supporters from the study.

#### 2.3.5. Peer Supporter Training

TEAM-MED peer supporters will attend two full training days delivered by qualified experts in dietary behaviour change and group facilitation skills. The training aims to equip peer supporters with knowledge (e.g., MD components, role of a peer supporter and behavioural strategies to support dietary change), confidence (e.g., practical experience in delivering the intervention resources) and skills (e.g., group facilitation and communication skills) to effectively deliver the peer support intervention. Peer support training will be interactive and informal, with emphasis on the provision of social support to encourage positive dietary change. An outline of peer supporter training components is shown in Table 2. Peer supporters will complete a knowledge questionnaire post-training, which will be used to determine individual competence to deliver the peer support intervention. A certificate will be given to those peer supporters who are successful in completing the training course (>75% questionnaire score). If competency to deliver the intervention is not achieved at this stage, further training will be offered to peer supporters via refresher day training and competency will be reassessed by administering the post-training questionnaire (as outlined above).

#### 2.3.6. Peer Supporter Support

Peer supporters will be provided with ongoing support from the study team during implementation of the intervention. A detailed training manual (‘TEAM-MED peer supporter manual’) will be provided to support delivery of the group intervention. The study team will promote ongoing communication between paired peer supporters to plan individual roles for group session delivery. Peer supporters will receive one scheduled telephone call from a researcher after individual group sessions to discuss any challenges or difficulties they may have experienced. In addition, all trained peer supporters will have an opportunity to meet as a group on at least one occasion during the intervention period, where informal discussion and sharing of experiences will be encouraged. Peer supporters will not be formally paid for their role but will receive reimbursement of travel costs incurred to deliver the intervention. They will also be provided with a pre-paid smart phone to facilitate communication with the study team, peer supporters and group participants. 

### 2.4. TEAM-MED Intervention Groups

#### 2.4.1. Group 1: Peer Support (*n* = 25)

A full description of the theory-based, tailored peer support intervention is published separately [30] and outlined below. Participants allocated to this intervention will be scheduled to attend a group programme delivered by two trained peer supporters comprising 11 group sessions over the 12-month period. Groups will involve up to 10 study participants who will meet in a convenient community setting, such as a private room in a community centre. Each group session is expected to last up to 2-h and will include a brief (10–15 min) MD and/or behavioural education component delivered by peer supporters and designed to provide a focus for group discussion. The group topics include: ‘health benefits of a MD’, ‘changing fat intake’, ‘eating more wholegrain’ and ‘eating a seasonal MD’. Practical food demonstrations (via food tasting sessions) are included in four group sessions. A personal weigh-in and blood pressure measurement will be available in each session, with feedback offered by a peer supporter. Participants will be supported with written MD educational materials, suggested meal plans, shopping lists and seasonal recipe books developed specifically for the study to support MD behaviour change. In addition, a personal workbook will be given to participants at the beginning of the group programme to facilitate dietary goal-setting and self-monitoring of personal dietary goals. Participants are encouraged to maintain contact with other group members and peer supporters between sessions (via telephone, text messaging and/or face-to-face meetings) to promote social support and group cohesion.

#### 2.4.2. Group 2: Dietitian-Led Support (*n* = 25)

The dietitian-led intervention is based on that reported in the PREDIMED trial and shown to be effective in achieving increased adherence to a MD [8]. At baseline, participants will attend an individual face-to-face 90-min motivational interview with a study dietitian. Personal MD dietary goals will be agreed and participants will receive written MD educational materials, suggested meal plans, shopping lists and seasonal recipes identical to those suggested to those in the other intervention groups. Participants will also be given key MD foods for daily consumption based on their personal preference for either:

- 50 mL extra virgin olive oil (EVOO) or,
- 30 g nuts (15 g walnuts, 7.5 g almonds and 7.5 g hazelnuts) or,
- Combination of both (25 mL EVOO plus 15 g nuts (8 g walnuts, 3.5 g almonds and 3.5 g hazelnuts))

Participants will be scheduled to attend a 2-h quarterly (3-, 6-, 9- and 12-months) structured group education session led by the dietitian, with up to four other study participants. The four group session topics are: ‘changing to a MD’, ‘enjoying fruit and vegetables’, ‘eating more wholegrain’ and ‘continuing to eat a MD’. Seasonal recipe books, suggested meal plans and shopping lists will be discussed at each session. The dietitian will then provide an individual 15-min progress review and feedback after each group session. Throughout the intervention period, and between group sessions, participants will have unlimited telephone/email contact with the dietitian for on-going support to optimise compliance with the intervention.

#### 2.4.3. Group 3: Minimal Support (Control) (*n* = 25)

Participants allocated to this group will receive the full TEAM-MED study written MD educational materials, suggested meal plans, shopping lists and seasonal recipe books at baseline. No further support will be provided. Participants will be offered an individual appointment with a researcher for personal MD advice at the end of the intervention period.

## 3. Results

### 3.1. Outcomes

TEAM-MED will evaluate feasibility of the peer support intervention, in comparison to different methods of providing dietary support, to change dietary behaviour toward a MD. Target behaviour change for the intervention is defined as a ≥ 3-point increase in MDS from baseline to 6 months (adoption) as this change is likely to be both achievable by the target population [15] and clinically important [40]. We will also evaluate change in MDS from 6 to 12 months i.e., maintenance of MD behavioural changes.

The variability data on change in MDS in response to the peer support intervention from baseline to 6 months will inform the sample size for a larger study. TEAM-MED will explore indicative effects of the intervention on CVD risk factors (e.g., body weight, blood pressure, fasting lipid profile, inflammatory markers) and markers of diabetes risk (blood glucose and glycated haemoglobin (HbA1c) levels) over the 12 months to inform the outcomes for a future definitive trial that will aim to assess clinical effectiveness of the peer support intervention for cardiometabolic health. Possible mediators of MD behaviour change in response to the intervention will also be explored as well as contextual factors that could influence implementation of the intervention or moderate the study outcomes.

TEAM-MED will determine recruitment and retention rates, evaluate data collection methods for outcomes and assess participant acceptability of the peer support intervention and acceptability of changing dietary behaviour towards a MD. The full methodology used for process evaluation will be presented in a separate manuscript. 

### 3.2. Study Measures

Study assessments will be conducted face-to-face at a dedicated research facility by a study researcher at baseline, 3-, 6- and 12-months. Each assessment visit will follow strict standardised operating procedures and last approximately 3.5 h. Table 3 outlines the study outcome measurements and data collection methods to be used at each specified time-point. These are discussed in more detail below. 

#### 3.2.1. Primary Outcome—MD Behaviour Change

A 14-item MDS questionnaire based on a similar questionnaire used in PREDIMED [8] and adapted to incorporate food choices in a Northern European population, will be used to determine MD adherence and ease of adoption of dietary behaviours consistent with a MD (see Table S2). Overall dietary intake will be assessed using a 4-day food record at each time-point. Participants will be given full instruction on how to complete the food record and asked to record all food/beverage items at the time of consumption. Furthermore, nutrient biomarkers will be used to assess MD compliance as discussed in Section 3.2.3.

#### 3.2.2. Clinical Measures

Blood pressure (mmHg) will be measured in the dominant arm, after a 5-min rest in a seated position, using a calibrated automated sphygmomanometer (Omron M5-1, OMRON Healthcare UK Ltd., Milton Keynes, UK). Three separate blood pressure measurements will be recorded over a five-minute period and summary systolic and diastolic blood pressure will be calculated from the second and third readings [41]. Anthropometric measurements will be made in light clothing without footwear using standard techniques. Standing height will be measured to the nearest 0.1 cm using a wall-mounted stadiometer. Body weight will be measured using a calibrated digital weighing scale (Tanita HS-301, Tanita, Yiewsley, UK) to the nearest 0.1 kg. Waist and hip circumference will be measured using a flexible tape and recorded to the nearest 0.1 cm.

#### 3.2.3. Biochemical Measures

A fasting blood sample will be obtained at each study visit using standard procedures by a trained phlebotomist. Samples will be centrifuged at 3000 rpm for 15 min within two hours of collection and stored in aliquots at −80 °C until analysis. A panel of nutrient biomarkers will be measured in plasma/serum samples to reflect the MDS food group targets and overall compliance with the MD in the intervention groups. Nutrient biomarkers will include: serum carotenoids and serum vitamin E by high performance liquid chromatography with diode array detection [42], plasma vitamin C by fluorimetric assay [43] and plasma fatty acids by gas chromatography [44,45]). A broad range of biochemical measures will be assessed to provide indicative effects of the intervention on cardiometabolic health and will include: fasting plasma glucose and 2-h plasma glucose (after a 75 g oral glucose load) measured using an automated glucose oxidase method using a Beckman Glucose Analyzer 2, as well as HbA1c and a fasting lipid profile measured using commercially available kits on an ILab-600 biochemical analyzer (Instrumentation Laboratory, Warrington, UK). A spot non-fasted urine sample will be obtained during the study visit for long term storage and future determination of nutritional and CVD biomarkers.

#### 3.2.4. Mediators of MD Behaviour Change

Several questionnaires will be administered at each study assessment time-point to explore potential mediators of MD behaviour change. Social support will be assessed, across dimensional scales, stress scales, different sources of support and types of support including informational, practical and emotional support, using a modified social support inventory questionnaire previously used in the Mediterranean Lifestyle Programme (MLP) intervention [46]. Problem-solving ability will be assessed using an adapted version of a problem-solving questionnaire also used in the MLP [20] (Dr. D. Toobert, personal communication). Additional questionnaires will be used to assess MD knowledge [47], barriers to MD consumption (based on qualitative research with target population [34] and self-efficacy to change dietary behaviour [48] adapted specifically to determine self-efficacy for change in MD behaviours for the current study.

#### 3.2.5. Intervention Moderators

Individual demographics, medication use and smoking status will be determined using a questionnaire developed for the study. Physical activity will be assessed using a validated Recent Physical Activity Questionnaire (RPAQ) [49,50]. Health beliefs will be investigated using a previously validated questionnaire based on components of the Health Belief Model [51] and modified for the present study. Several questionnaires will also be used to assess psychosocial well-being as potential moderators of MD behaviour change. The Rand 36-item (SF-36) validated questionnaire [52] will be used to assess self-reported quality of life and the ED-5Q-3L validated questionnaire will be used to determine generic health status [53]. A 34-item questionnaire (adapted for use in our population) will be used to report diet-related quality of life (adapted from [54]). Self-reported mood will be determined using the Positive and Negative Affect Schedule (PANAS) [55] questionnaire comprising two 10-item mood scales. Self-esteem will be assessed using a validated 10-item questionnaire [56]. 

#### 3.2.6. Evaluation of Recruitment and Study Attrition Rates

Study records completed by the research team will provide quantitative data on participant recruitment rates from different sources, recruitment of peer supporters, withdrawal from the study and reported reasons for withdrawal. Data will be used to evaluate recruitment strategies and estimate attrition and retention rates. 

### 3.3. Power and Sample Size

A primary objective of TEAM-MED is to determine change in MDS in response to the peer support intervention from the target population and generate variability data to estimate sample size for a definitive trial. However, we were able to calculate likely power for the study using data generated in a previous intervention trial of patients with existing CVD [15]. Based on the MDS changes in the behavioural counselling group of that trial, that showed standard deviations of 1.3 from baseline to 6 months, a study of 25 subjects per group would have approximately 90% power to detect a difference between treatment groups in mean MDS change from baseline to 6 months of 1.4 units. This assumes a 30% dropout rate during the study period. Corresponding standard deviation for vitamin C change (which is an indicator of fruit and vegetable intake and therefore may be one biomarker associated with adherence to the MD) is 13.9 µmol/L and the difference in mean vitamin C change that could be detected with approximately 90% power is 15 µmol/L. Given that TEAM-MED is evaluating a different intervention to encourage MD adherence than that used previously, and in a different population, the data generated here will provide a more reliable sample size calculation for a definitive study.

### 3.4. Statistical Analyses

Study data will be handled according to the Data Protection Act and will be anonymised and archived in password-protected study databases. Data entry will be performed by trained researchers. Data will be screened, cleaned and validated by the study team by performing summary statistical checks for frequency distributions, missing data and outlier detection. The final dataset for analysis will be approved by the study statistician. Data analysis will be conducted using SPSS version 23.0. Descriptive statistics will be provided for each of the three intervention groups at baseline and include demographic, dietary, lifestyle and clinical information. Continuous variables will be summarized using Mean and SD; categorical variables will be summarised using counts and percentages. To determine intervention group differences in repeated MDS and other end-point measures generated by the study, we will use statistical techniques appropriate for longitudinal data analysis e.g., linear mixed effects models. Initial examination of the correlation structure of the repeated measures will help identify significant interactions between potential confounders and guide model fitting. Linear models will be adjusted for covariates where appropriate and both unadjusted and adjusted models will be presented. The difference in means will provide an estimate of the effect of intervention and 95% confidence limits will be calculated to indicate its precision. If appropriate, measurements will be logarithmically transformed prior to analysis and interpretation will be made in ratio terms on the original scale. Statistical significance will be set at *p* ≤ 0.05. Process evaluation data analysis will be presented in a separate manuscript. 

## 4. Discussion

Supporting adoption and maintenance of a healthy diet such as the MD, which has been shown to effectively reduce the risk of disease, should be a high public health priority for disease prevention. Most trials evaluating the health impacts of a MD have been conducted in Mediterranean countries. One exception is the MedLey study in older Australians that demonstrated improved CVD risk factors (blood pressure, triglycerides, and F2-isoprostanes) as well as greater flow-mediated dilatation [57,58] after a 6-month MD intervention. Dietary behaviour change was achieved in this study using intensive methods: dietetic consultations and motivational interviewing at 3 and 6 months and every fortnight in between, provision of written educational materials, sample menus and recipes, a daily food checklist to help track compliance with the diet and provision of key foods [59]. While these findings indicate that behaviour change towards a MD is achievable with a health professional led intervention in a non-Mediterranean population, there is still much to learn regarding the most cost-effective approaches to support MD behaviour change [60], and particularly, interventions to support longer-term maintenance of newly adopted MD behaviours in non-Mediterranean countries. This paper describes the protocol for the TEAM-MED trial to explore feasibility of a developed peer support intervention for dietary behaviour change towards a MD, compared to another more intensive method of support, in Northern Europeans at high CVD risk. The intervention has been developed using a theory-based approach and is tailored to the needs of the target group to promote dietary behaviour change. TEAM-MED benefits from including a range of objective and subjective measures of diet adherence over 12 months, which will help to determine adoption and maintenance of dietary behaviours consistent with a MD. TEAM-MED also incorporates a comprehensive evaluation plan to determine acceptability and fidelity of intervention implementation and will explore potential important mediators of dietary change in the population and these methods will be more comprehensively described in a separate paper. 

## 5. Conclusions

Findings from this study will generate important data for acceptability and feasibility of the peer support intervention and the ease of adoption of MD in an ‘at risk’ group in a Northern European population with low MD adherence. Ultimately, data generated here will be used to inform the design of a larger scale RCT, where the clinical efficacy and cost-effectiveness of the peer support intervention will be tested. 

## Figures and Tables

**Table 1:** Behaviour Change Techniques used to encourage dietary change towards a Mediterranean diet.

TEAM-MED Intervention Groups	BCTs (*n*)	BCT Label (from BCT Taxonomy ^1^ or CALO-RE ^2^)	BCT Definition	Example of BCT Delivery in the Intervention Groups
**Group 1: Peer support**	18	Provide information on consequences of behaviour in general ^2^	Information about the relationship between the behaviour and its possible or likely consequences in the general case, usually based on epidemiological data, and not personalised for the individual	Peer supporters show a short video clip to group members demonstrating the health effects of a MD
		Provide normative behaviour about others’ behaviour ^2^	Involves providing information about what other people are doing i.e., indicates that a particular behaviour or sequence of behaviours is common or uncommon amongst the population or amongst a specified group—presentation of case studies of a few others is not normative information.	Peer supporters provide information about current MD adherence in Northern European populations
		Goal setting (Behaviour) ^1,2^	Set or agree on a goal defined in terms of the behaviour to be achieved	Peer supporters support members to set MD goals at each group session based on the session topic
		Goal setting (outcome) ^1,2^	Set or agree on a goal defined in terms of a positive outcome of wanted behaviour	Group members are encouraged within their personal planners to define what they want to achieve by taking part in the peer support groups, e.g., target weight loss, or decreasing to target blood pressure level etc.
		Action planning ^1,2^	Prompt detailed planning of performance of the behaviour	Peer supporters support members to set MD goals that are easy to measure, something that can be achieved, small and meaningful (i.e., SMART goals) at each group session
		Barrier identification/problem solving ^1,2^	Analyse, or prompt the person to analyse, factors influencing the behaviour and generate or select strategies that include overcoming barriers and/or increasing facilitators	Peer supporters facilitate group discussion to identify barriers/challenges in achieving personal MD goals and assist members to select the best strategies to overcome these
		Set graded tasks ^1,2^	Set easy-to-perform tasks, making them increasingly difficult, but achievable, until behaviour is performed	Increasing adherence to a MD is broken down into smaller tasks within written materials, e.g., food swaps are listed separately for each major MD component
		Prompt review of behavioural goals ^2^	Involves a review or analysis of the extent to which previously set behavioural goals were achieved	Each group session will provide an opportunity for general progress review in terms of behaviour
		Prompt self-monitoring of behaviour ^2^	The person is asked to keep a record of specified behaviour(s) as a method for changing behaviour.	Group members are given personal planners to monitor their daily/weekly progress in achieving set MD goals and to allow them to record any barriers/challenges they experience
		Prompt self-monitoring of behavioural outcome ^2^	The person is asked to keep a record of specified measures expected to be influenced by the behaviour change	Group members are encouraged to log and monitor their weight, blood pressure etc. in personal planners
		Provide information on when and where to perform the behaviour ^2^	Involves telling the person about when and where they might be able to perform the behaviour	Recipe books and written information provide information regarding different meals, and also eating out as well as eating in the home
		Provide instruction on how to perform behaviour ^1,2^	Involves telling the person how to perform behaviours, either verbally or in written form.	Peer supporters provide group members with a booklet and a visual guide (MD food pyramid) to provide instruction on the types and proportions of food components in a MD
		Model/demonstrate the behaviour ^1,2^	Provide an observable sample of the performance of the behaviour, directly in person or indirectly	Peer supporters show a short video clip to group members demonstrating preparation and consumption of a MD on a budget and food tasting sessions form part of peer group meetings
		Use of follow-up prompts ^2^	Intervention components are gradually reduced in intensity, duration and frequency over time, e.g., letters or telephone calls instead of face-to-face and/or provided at longer time intervals	Group sessions decrease in frequency after six months
		Plan social support/social change ^2^	Involves prompting the person to plan how to elicit social support from other people to help him/her achieve their target behaviour/outcome.	Group members are encouraged to support and contact each other between group sessions
		Relapse prevention/coping planning ^2^	This relates to planning how to maintain behaviour that has been changed. The person is prompted to identify in advance situations in which the changed behaviour may not be maintained and develop strategies to avoid or manage those situations	One group session (session nine) is dedicated to maintenance of dietary change and relapse prevention
		Biofeedback ^1^	Provide feedback about the body using an external monitoring device as part of a behaviour change strategy	Peer supporters offer individual feedback on blood pressure and weight measurements at each group session
		Social support ^1^	Advise on, arrange or provide social support or non-contingent praise or reward for performance of the behaviour	Peer supporters and group members provide positive encouragement and support to each-other to adopt new MD behaviours
**Group 2: Dietitian-led support**	20	Motivational interviewing ^2,^*	This is a clinical method including a specific set of techniques involving prompting the person to engage in change talk in order to minimise resistance and resolve ambivalence to change	Participants attend individual motivational interviewing delivered by a trained dietitian
		Provide information on consequences of behaviour in general ^2^	Information about the relationship between the behaviour and its possible or likely consequences in the general case, usually based on epidemiological data, and not personalised for the individual	Dietitian shows a short video clip demonstrating the health effects of a MD within the structured group education sessionHealth consequences of MD also detailed in educational material
		Provide information on consequences of behaviour to the individual ^2^	Information about the benefits and costs of action or inaction to the individual or tailored to a relevant group based on that individual’s characteristics	Discussion of dietary change to encourage adherence to a MD occurs specifically based on individual’s current level of adherence, and with knowledge of their CVD risk score
		Provide normative behaviour about others’ behaviour ^2^	Involves providing information about what other people are doing i.e., indicates that a particular behaviour or sequence of behaviours is common or uncommon amongst the population or amongst a specified group—presentation of case studies of a few others is not normative information.	Dietitian provides information about current MD adherence in Northern European populations
		Goal setting (Behaviour) ^1,2^	Set or agree on a goal defined in terms of the behaviour to be achieved	Discussed during motivational interview with dietitian
		Goal setting (outcome) ^1,2^	Set or agree on a goal defined in terms of a positive outcome of wanted behaviour	Discussed during motivational interview with dietitian
		Action planning ^1,2^	Prompt detailed planning of performance of the behaviour	Discussed during motivational interview with dietitian
		Set graded tasks ^1,2^	Set easy-to-perform tasks, making them increasingly difficult, but achievable, until behaviour is performed	Increasing adherence to a MD is broken down into smaller tasks within educational materials, e.g., food swaps are listed separately for each major MD component Reinforced by dietitian in group education session and in individual session
		Provide information on when and where to perform the behaviour ^2^	Involves telling the person about when and where they might be able to perform the behaviour	Recipe books and written information provide information regarding different meals, and also eating out as well as eating in the home
		Provide instruction on how to perform behaviour ^1,2^	Involves telling the person how to perform behaviours, either verbally or in written form.	Practical support around shopping lists, recipes, food storage and preparation given within educational material
		Barrier identification/problem solving ^1,2^	Analyse, or prompt the person to analyse, factors influencing the behaviour and generate or select strategies that include overcoming barriers and/or increasing facilitators	Discussion about challenges in meeting dietary goals and developing strategies to overcome these in group education session
		Prompt review of behavioural goals ^2^	Involves a review or analysis of the extent to which previously set behavioural goals were achieved	Individual progress review and feedback provided at end of group education session
		Prompt self-monitoring of behaviour ^2^	The person is asked to keep a record of specified behaviour(s) as a method for changing behaviour	Plan to monitor progress in achieving goals developed during motivational interview
		Provide feedback on performance ^2^	This involves providing the participant with data about their own recorded behaviour	Individual progress review and feedback provided at end of group education session
		Model/demonstrate the behaviour ^1,2^	Provide an observable sample of the performance of the behaviour, directly in person or indirectly	Video clip demonstrating preparation and consumption of a MD on a budget shown in group education session
		Plan social support/social change ^2^	Involves prompting the person to plan how to elicit social support from other people to help him/her achieve their target behaviour/outcome.	Discussion of support around family structure and food purchasing/preparation included in motivational interview and individual progress review. Social support encouraged through participation in group education session with other participants.
		Relapse prevention/coping planning ^2^	This relates to planning how to maintain behaviour that has been changed. The person is prompted to identify in advance situations in which the changed behaviour may not be maintained and develop strategies to avoid or manage those situations.	Challenging situations discussed during both motivational interview and group education session
		Commitment ^1^	Ask the person to affirm or reaffirm statements indicating commitment to change the behaviour	Affirmation of personal dietary goals sought during motivational interview
		Credible source ^1^	Present verbal or visual communication from a credible source in favour of or against the behaviour	Both motivational interview and group education session delivered by trained dietitian
		Adding objects to the environment ^1^	Add objects to the environment in order to facilitate performance of the behaviour	Key foods delivered to participants
**Group 3: Minimal support**	4	Provide information on consequences of behaviour in general ^2^	Information about the relationship between the behaviour and its possible or likely consequences in the general case, usually based on epidemiological data, and not personalised for the individual	Health consequences of MD detailed in educational material
		Set graded tasks ^1,2^	Set easy-to-perform tasks, making them increasingly difficult, but achievable, until behaviour is performed	Increasing adherence to a MD is broken down into smaller tasks within written materials, e.g., food swaps are listed separately for each major MD component
		Provide information on when and where to perform the behaviour ^2^	Involves telling the person about when and where they might be able to perform the behaviour	Recipe books and written information provide information regarding different meals, and also eating out as well as eating in the home
		Provide instruction on how to perform behaviour ^1,2^	Involves telling the person how to perform behaviours, either verbally or in written form.	Practical support around shopping lists, recipes, food storage and preparation given within educational material

BCT = Behaviour Change Techniques derived from published taxonomies (see ref. [36] ^1^, [37] ^2^); * Motivational Interview is delivered by a trained Dietitian and centred on the individual therefore listed BCTs are considered core within the intervention but additional BCTs are likely to be used to elicit dietary behaviour change at the individual level. TEAM-MED: Trial to Encourage Adoption and Maintenance of a MEditerranean Diet. MD: Mediterranean diet.

**Table 2:** Overview of Peer supporter training programme delivered over two consecutive days.

Day	Training Components
One (7 h)	Mediterranean Diet food components, health benefits and pyramid model Role of a peer supporter Group intervention format, content and introduction to resources Practical dietary advice to overcome barriers to eating a Mediterranean diet Weight and blood pressure measurement workshop including provision of feedback Maintaining study documentation for group sessions
Two (7 h)	Social support in a group setting to encourage dietary change Group facilitation skills Delivering group resources Handling difficult group situations

**Table 3:** TEAM-MED outcome measures and data collection methods.

Outcome	Domain to Be Measured	Data Collection Method(s)	Baseline	3 Months	6 Months	12 Months
**Diet**	MD adherence	14-item MDS questionnaire [8] ^2^	√	√	√	√
	Dietary intake	Food record (4-day)	√	√	√	√
**Clinical & biomarker**	Nutritional biomarkers	Venepuncture (fasting blood sample) OGTT	√	√	√	√
	Impaired glucose tolerance		√	√	√	√
	HbA1_C_		√	√	√	√
	Blood pressure	Clinic measured [41]	√	√	√	√
	Nutrition and CVD markers	Urine sample (spot fasting sample)	√	√	√	√
	Weight	Digital scales	√	√	√	√
	Height	Stadiometer	√	√	√	√
	Waist circumference	Flexible tape	√	√	√	√
**Mediators of diet behaviour change**	MD knowledge	Nutrition knowledge questionnaire [47] ^2^	√	√	√	√
	Readiness to change	Stage of dietary change questionnaire [38]	√	√	√	√
	Perceived barriers to MD	Eating habits questionnaire ^1^	√	√	√	√
	Self-efficacy	Questionnaire [48]	√	√	√	√
	Social support	Questionnaire [46] ^2^	√	×	√	√
	Problem solving ability	Questionnaire [20] ^2^	√	√	√	√
**Intervention Moderators**	Physical activity	RPAQ questionnaire [49,50]	√	√	√	√
	Smoking, alcohol, medication use	Questionnaire ^1^	√	√	√	√
	Health beliefs	Health belief questionnaire [51] ^2^	√	√	√	√
	Health-related Quality of Life	SF-36 [52]; EQ-5D-3L [53]	√	√	√	√
	Diet-related Quality of life	Questionnaire [54] ^2^	√	√	√	√
	Mood	Questionnaire [55]	√	√	√	√
	Self-esteem	Self-esteem questionnaire [56]	√	√	√	√

^1^ Developed for TEAM-MED; ^2^ Modified for TEAM-MED. TEAM-MED: Trial to Encourage Adoption and Maintenance of a MEditerranean Diet. MD: Mediterranean diet. MDS: Mediterranean Diet Score. OGTT: Oral Glucose Tolerance Test. CVD: Cardiovascular disease. RPAQ: Recent Physical Activity Questionnaire.

## Supplementary Materials

The following are available online at http://www.mdpi.com/1660-4601/15/6/1130/s1, Table S1: SPIRIT checklist, Table S2: TEAM-MED Mediterranean Diet Score screener.
