# Supplementary material for: Trial to Encourage Adoption and Maintenance of a Mediterranean Diet (TEAM-MED): Protocol for a Randomised Feasibility Trial of a Peer Support Intervention for Dietary Behaviour Change in Adults at High Cardiovascular Disease Risk
Source: Int J Environ Res Public Health. 2018 May 31;15(6):1130. doi: 10.3390/ijerph15061130 (PMC6025132; doi:10.3390/ijerph15061130)
Supplement: Supplementary file 1 [file ijerph-15-01130-s001.pdf]

SPIRIT

STANDARD PROTOCOL ITEMS: RECOMMENDATIONS FOR INTERVENTIONAL TRIALS

SPIRIT 2013 Checklist: Recommended items to address in a clinical trial protocol and related documents*

Section/item	Item No	Description	Addressed on page number
**Administrative information**			
Title	1	Descriptive title identifying the study design, population, interventions, and, if applicable, trial acronym	_______1______
Trial registration	2a	Trial identifier and registry name. If not yet registered, name of intended registry	_____4_______
	2b	All items from the World Health Organization Trial Registration Data Set	______4_______
Protocol version	3	Date and version identifier	_______4______
Funding	4	Sources and types of financial, material, and other support	_______14______
Roles and responsibilities	5a	Names, affiliations, and roles of protocol contributors	_____14___
	5b	Name and contact information for the trial sponsor	_______14______
	5c	Role of study sponsor and funders, if any, in study design; collection, management, analysis, and interpretation of data; writing of the report; and the decision to submit the report for publication, including whether they will have ultimate authority over any of these activities	_____14________
	5d	Composition, roles, and responsibilities of the coordinating centre, steering committee, endpoint adjudication committee, data management team, and other individuals or groups overseeing the trial, if applicable (see Item 21a for data monitoring committee)	__N/A__________

**Introduction**			
Background and rationale	6a	Description of research question and justification for undertaking the trial, including summary of relevant studies (published and unpublished) examining benefits and harms for each intervention	____2-3_____
	6b	Explanation for choice of comparators	____8-9, ref 30___
Objectives	7	Specific objectives or hypotheses	____3___
Trial design	8	Description of trial design including type of trial (eg, parallel group, crossover, factorial, single group), allocation ratio, and framework (eg, superiority, equivalence, noninferiority, exploratory)	____4________
**Methods: Participants, interventions, and outcomes**			
Study setting	9	Description of study settings (eg, community clinic, academic hospital) and list of countries where data will be collected. Reference to where list of study sites can be obtained	______6____
Eligibility criteria	10	Inclusion and exclusion criteria for participants. If applicable, eligibility criteria for study centres and individuals who will perform the interventions (eg, surgeons, psychotherapists)	_____6-7______
Interventions	11a	Interventions for each group with sufficient detail to allow replication, including how and when they will be administered	_____7-8____
	11b	Criteria for discontinuing or modifying allocated interventions for a given trial participant (eg, drug dose change in response to harms, participant request, or improving/worsening disease)	___N/A________
	11c	Strategies to improve adherence to intervention protocols, and any procedures for monitoring adherence (eg, drug tablet return, laboratory tests)	__7-8___
	11d	Relevant concomitant care and interventions that are permitted or prohibited during the trial	____N/A_
Outcomes	12	Primary, secondary, and other outcomes, including the specific measurement variable (eg, systolic blood pressure), analysis metric (eg, change from baseline, final value, time to event), method of aggregation (eg, median, proportion), and time point for each outcome. Explanation of the clinical relevance of chosen efficacy and harm outcomes is strongly recommended	______9-11___
Participant timeline	13	Time schedule of enrolment, interventions (including any run-ins and washouts), assessments, and visits for participants. A schematic diagram is highly recommended (see Figure)	_Figure 1___

Sample size	14	Estimated number of participants needed to achieve study objectives and how it was determined, including clinical and statistical assumptions supporting any sample size calculations	_____13________
Recruitment	15	Strategies for achieving adequate participant enrolment to reach target sample size	_____8-_9_

Methods: Assignment of interventions (for controlled trials)

Allocation:			
Sequence generation	16a	Method of generating the allocation sequence (eg, computer-generated random numbers), and list of any factors for stratification. To reduce predictability of a random sequence, details of any planned restriction (eg, blocking) should be provided in a separate document that is unavailable to those who enrol participants or assign interventions	______4_______
Allocation concealment mechanism	16b	Mechanism of implementing the allocation sequence (eg, central telephone; sequentially numbered, opaque, sealed envelopes), describing any steps to conceal the sequence until interventions are assigned	______4_______
Implementation	16c	Who will generate the allocation sequence, who will enrol participants, and who will assign participants to interventions	____4_________
Blinding (masking)	17a	Who will be blinded after assignment to interventions (eg, trial participants, care providers, outcome assessors, data analysts), and how	_____4________
	17b	If blinded, circumstances under which unblinding is permissible, and procedure for revealing a participant's allocated intervention during the trial	_____4________

Methods: Data collection, management, and analysis

Data collection methods	18a	Plans for assessment and collection of outcome, baseline, and other trial data, including any related processes to promote data quality (eg, duplicate measurements, training of assessors) and a description of study instruments (eg, questionnaires, laboratory tests) along with their reliability and validity, if known. Reference to where data collection forms can be found, if not in the protocol	____9-12__
	18b	Plans to promote participant retention and complete follow-up, including list of any outcome data to be collected for participants who discontinue or deviate from intervention protocols	____9-12_____

Data management	19	Plans for data entry, coding, security, and storage, including any related processes to promote data quality (eg, double data entry; range checks for data values). Reference to where details of data management procedures can be found, if not in the protocol	__13___
Statistical methods	20a	Statistical methods for analysing primary and secondary outcomes. Reference to where other details of the statistical analysis plan can be found, if not in the protocol	____13_____
	20b	Methods for any additional analyses (eg, subgroup and adjusted analyses)	_______N/A_____
	20c	Definition of analysis population relating to protocol non-adherence (eg, as randomised analysis), and any statistical methods to handle missing data (eg, multiple imputation)	__N/A__________

Methods: Monitoring

Data monitoring	21a	Composition of data monitoring committee (DMC); summary of its role and reporting structure; statement of whether it is independent from the sponsor and competing interests; and reference to where further details about its charter can be found, if not in the protocol. Alternatively, an explanation of why a DMC is not needed	__N/A__________
	21b	Description of any interim analyses and stopping guidelines, including who will have access to these interim results and make the final decision to terminate the trial	__N/A__________
Harms	22	Plans for collecting, assessing, reporting, and managing solicited and spontaneously reported adverse events and other unintended effects of trial interventions or trial conduct	__N/A__________
Auditing	23	Frequency and procedures for auditing trial conduct, if any, and whether the process will be independent from investigators and the sponsor	__N/A__________

Ethics and dissemination

Research ethics approval	24	Plans for seeking research ethics committee/institutional review board (REC/IRB) approval	_4___________
Protocol amendments	25	Plans for communicating important protocol modifications (eg, changes to eligibility criteria, outcomes, analyses) to relevant parties (eg, investigators, REC/IRBs, trial participants, trial registries, journals, regulators)	____4_________

Consent or assent	26a	Who will obtain informed consent or assent from potential trial participants or authorised surrogates, and how (see Item 32)	____4_________
	26b	Additional consent provisions for collection and use of participant data and biological specimens in ancillary studies, if applicable	___4_________
Confidentiality	27	How personal information about potential and enrolled participants will be collected, shared, and maintained in order to protect confidentiality before, during, and after the trial	_On request
Declaration of interests	28	Financial and other competing interests for principal investigators for the overall trial and each study site	____14_____
Access to data	29	Statement of who will have access to the final trial dataset, and disclosure of contractual agreements that limit such access for investigators	On request
Ancillary and post-trial care	30	Provisions, if any, for ancillary and post-trial care, and for compensation to those who suffer harm from trial participation	On request
Dissemination policy	31a	Plans for investigators and sponsor to communicate trial results to participants, healthcare professionals, the public, and other relevant groups (eg, via publication, reporting in results databases, or other data sharing arrangements), including any publication restrictions	___N/A_________
	31b	Authorship eligibility guidelines and any intended use of professional writers	______N/A______
	31c	Plans, if any, for granting public access to the full protocol, participant-level dataset, and statistical code	_______N/A_____
**Appendices**			
Informed consent materials	32	Model consent form and other related documentation given to participants and authorised surrogates	On request
Biological specimens	33	Plans for collection, laboratory evaluation, and storage of biological specimens for genetic or molecular analysis in the current trial and for future use in ancillary studies, if applicable	_____10___

*It is strongly recommended that this checklist be read in conjunction with the SPIRIT 2013 Explanation & Elaboration for important clarification on the items. Amendments to the protocol should be tracked and dated. The SPIRIT checklist is copyrighted by the SPIRIT Group under the Creative Commons "Attribution-NonCommercial-NoDerivs 3.0 Unported" license.

Table S2. TEAM-MED: MEDITERRANEAN DIET SCORE (MDS)

Questions	Self-reported	Criteria for 1 point	Point?
1. Do you use olive oil or rapeseed oil as your main cooking fat?		Yes	
2. Do you use olive or rapeseed oil-based spreads (e.g. Golden olive, Olivio, Bertolli)?		Yes	
3. How much olive/ rapeseed oil do you consume in a given day? Including oil used for frying, salads, out-of-house meals etc (in tablespoons)<br><br>How much olive or rapeseed oil-based spread do you consume in a given day? (in teaspoons)		≥4 tbsp oil / day<br><br>**and/or**<br><br>3 tsp spread/ day	
4. How many portions of fruit (including natural fruit juices) do you consume per day? (1 portion = 1 apple/banana (80g), small glass juice (150ml)		≥2 portions/ day	
5. How many vegetable servings do you consume per day? Including raw/ cooked vegetables, salad but not including potatoes (1 serving: 3 tablespoons/80g)		≥3 portions/ day	
6. How many servings of legumes (peas, beans and lentils including kidney beans, baked beans, chickpeas, red lentils etc) do you consume per week? (1 serving :3 tablespoons/ 80g)		≥3 servings/ week	
7. How many servings of red meat including beef, pork, lamb and minced beef do you consume per week? (1 serving: medium portion/ 100–150 g)		≤2 servings /week	
8. How many servings of processed meat including ham, bacon, sausages, meat pies and other meat products etc.) do you consume per week? (1 serving: medium portion/ 100–150 g)		≤1 serving /week	
9. How many servings of chicken/ turkey do you consume per week? (1 serving: medium portion/ 100-150g)		2 servings /week	
10. How many servings of fish (tuna, cod, haddock, salmon, mackerel, herring, and sardines etc, including tinned varieties, excluding crumbed or battered fish) or shellfish do you consume per week? (1 serving: 1 fillet/small fish or 140g)		≥3 servings /week	
11. Do you preferentially consume wholegrain bread and/ or cereal and/ or rice and/ or pasta instead of non-wholegrain (white) varieties?		Yes	
12. How many servings of natural nuts do you consume per week? (1 serving: 1 small handful/ 30 g)		≥3 servings /week	
13. How many times per week do you consume sweet foods (including biscuits, buns, pastries, chocolate, sweets, sweet or carbonated beverages or desserts)?		≤3 times/ week	
14. How often would you consume up to 3 small glasses of wine or equivalent other alcoholic beverages per week? (1 small glass:125ml)		1-3 glasses or equivalent ≥ 3 days/ week	
